# EP300 mutation is associated with tumor mutation burden and promotes antitumor immunity in bladder cancer patients

**DOI:** 10.18632/aging.102728

**Published:** 2020-02-03

**Authors:** Gongmin Zhu, Lijiao Pei, Yuan Li, Xin Gou

**Affiliations:** 1 Department of Urology, The First Affiliated Hospital of Chongqing Medical University, Chongqing, China; 2 The State Key Laboratory of Biotherapy, West China Hospital, Sichuan University, Chengdu, China; 3 Department of Cardiothoracic Surgery, The First Affiliated Hospital of Chongqing Medical University, Chongqing, China

**Keywords:** bladder cancer, *EP300*, tumor mutation burden, tumor-infiltrating immune cells, prognosis

## Abstract

Bladder cancer is a leading cause of morbidity and mortality worldwide. Currently, immunotherapy has become a worthwhile therapy for bladder cancer. Tumor mutation burden (TMB) has been regarded as the most prevalent biomarker to predict immunotherapy. Bladder cancer is reported to have the third highest mutation rate. However, whether these gene mutations are related to TMB and immune response remain unknown. In this study, we downloaded somatic mutation data of bladder cancer from The Cancer Genome Atlas (TCGA) and International Cancer Genome Consortium (ICGC) datasets, and found 11 frequently mutated genes were covered by both two cohorts including FGFR3, TTN, XIRP2, CREBBP, PIK3CA, TP53, MUC16, EP300 (E1A binding protein P300), ARID1A, ERBB2, and KDM6A. Among them, EP300 mutation was associated with higher TMB and indicated a favorable clinical prognosis. Furthermore, based on Gene set enrichment analysis (GSEA) and CIBERSORT algorithm, we observed that EP300 mutation upregulated signaling pathways involved in immune system and enhanced antitumor immune response. In conclusion, EP300 is frequently mutated in bladder cancer, and its mutation is associated with increased TMB and promotes antitumor immunity, which may serve as a biomarker to predict immune response.

## INTRODUCTION

Worldwide, bladder cancer accounts for approximately 3.0% of all malignancies. In 2018, an estimated 549,393 bladder cancer cases were identified and contributed to 199,922 deaths [1]. Although approximately 70% of bladder cancer patients present with the non-muscle invasive type, as many as half of them experience disease recurrence after receiving transurethral resection, and up to quarter of them progress to muscle-invasive bladder cancer (MIBC) after repeated recurrences [2]. Moreover, approximately 30% of patients have muscle invasive disease at the time of initial diagnosis, and radical cystectomy is the current standard of care [3]. Nevertheless, even after surgery, approximately 50% of patients develop metastases [4]. Over the past years, systemic chemotherapy using cisplatin-based regimens has been the predominant treatment for metastatic urothelial carcinoma (mUC). However, approximately 50% of patients with mUC are ineligible to receive cisplatin due to severe adverse effects [5]. Therefore, for patients for whom first-line chemotherapy is not successful, there is an urgent need to find a novel treatment option to improve clinical outcomes.

Currently, with the emergence of immune checkpoint inhibitors targeting programmed cell death protein and its ligand (PD-1/PD-L1), immunotherapy has revolutionized anti-cancer therapy. Bladder cancer has been recognized as an immunoresponsive tumor, and immunotherapy has been reported to be a worthwhile neoadjuvant therapy for treatment of MIBC [6, 7]. However, only a minority of patients benefit from these immunotherapies, and fewer achieve a durable response [8]. Therefore, understanding the molecular determinants of immunotherapeutic responsiveness is one of critical challenges in bladder cancer. Emerging studies have explored the correlation between tumor mutation burden (TMB) and immunotherapy response [9, 10]. Accumulation of somatic mutations is one of the leading cause of tumorigenesis and contributes to the expression of neoantigens [11]. Neoantigens can activate CD8^+^ CTLs to act against tumors by recognizing target antigens presented on tumor cells [12]. Previous studies have reported that a high TMB predicts a better outcome in non-small cell lung cancer and melanoma [13, 14]. Meanwhile, TMB has also been regarded as a predictive biomarker of tumor behavior and immunological response [9, 15]. Previous study has demonstrated that bladder cancer has the third highest mutation prevalence and is highly antigenic [16]. However, whether these gene mutations are related to TMB and immune response in bladder cancer remains unclear.

In this study, we first identified somatic mutations in bladder cancer patients from US and China using The Cancer Genome Atlas (TCGA) dataset and International Cancer Genome Consortium (ICGC) dataset. Then, we found the common mutant genes in the two cohorts, and further explored the association of these gene mutations with TMB and prognosis. Finally, we investigated whether gene mutations are associated with immune response. Findings emerged from this study may identify a novel biomarker for TMB and suggest a potential immunotherapy for bladder cancer patients.

## RESULTS

### Landscape of somatic mutations in bladder cancer

**Figure 1:**
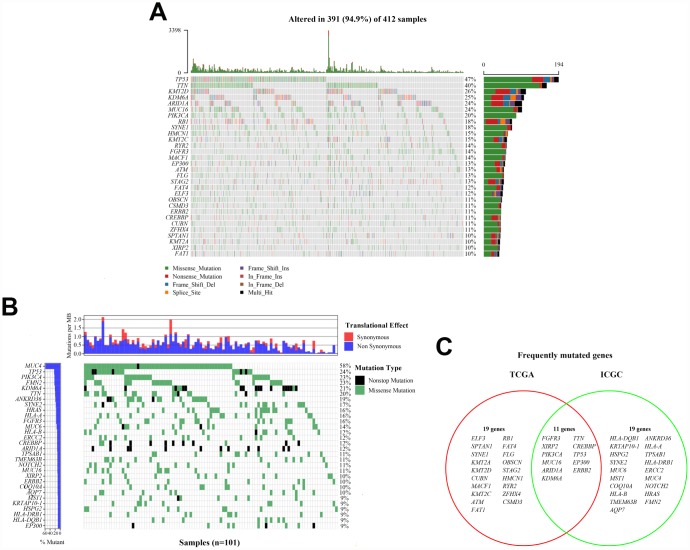
**Landscapes of frequently mutated genes in bladder cancer.** (**A**) Oncoplot depicts the frequently mutated genes in bladder cancer from TCGA cohort. The left panel shows mutation frequency, and genes are ordered by their mutation frequencies. The bottom panel presents different mutation types. (**B**) Waterfall plot displays the frequently mutated genes in bladder cancer from ICGC cohort. The left panel shows the genes ordered by their mutation frequencies. The right panel presents different mutation types. (**C**) Venn diagram of frequently mutated genes covered by both TCGA and ICGC cohorts.

We defined 30 frequently mutated genes in American bladder cancer samples from TCGA cohort, and the five most frequently mutated genes were *TP53* (47%), *TTN* (40%), *KMT2D* (26%), *KDM6A* (25%), and *ARID1A* (24%) (Figure 1A). Meanwhile, we also defined 30 frequently mutated genes in Chinese bladder cancer samples from ICGC cohort, and the five most frequently mutated genes were *MUC4* (58%), *TP53* (24%), *FMN2* (23%), *PIK3CA* (23%), and *KDM6A* (21%) (Figure 1B). Interestingly, we observed that some genes frequently mutated in both American and Chinese patients. Therefore, we performed comparative analysis of 30 frequently mutated genes between TCGA and ICGC cohorts of bladder cancer. 11 frequently mutated genes reported by TCGA cohort were covered by ICGC cohort, including *FGFR3*, *TTN*, *XIRP2*, *CREBBP*, *PIK3CA*, *TP53*, *MUC16*, *EP300*, *ARID1A*, *ERBB2*, and *KDM6A* (Figure 1C). Then, we focused on these common mutated genes in subsequent analysis.

### *EP300* mutation associated with TMB and survival prognosis

**Figure 2:**
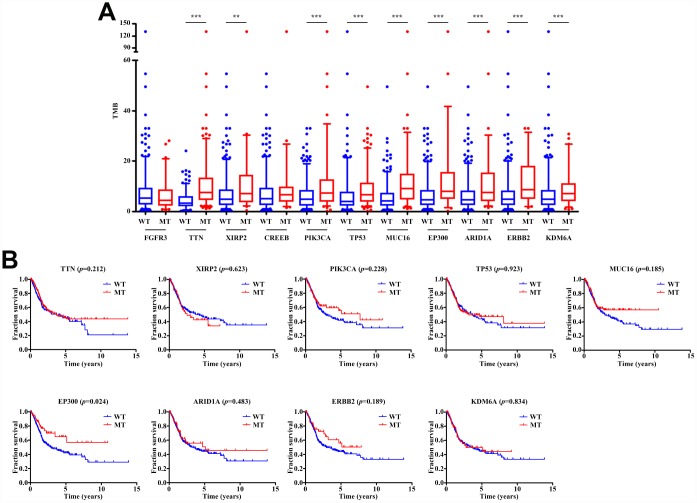
**Gene mutations are associated with TMB and clinical prognosis.** (**A**) Most gene mutations are associated with a higher TMB. ** *p*<0.01; *** *p*<0.001. (**B**) Kaplan-Meier survival analysis of patients with gene mutations. Only patients with complete clinical information were included (n=402). The *p-*value is marked in each graph. WT, wild type; MT, mutant type.

The TMB score in bladder cancer is ranging from 0.02 to 130 per Mb with a median of 5.06 per Mb. Among common mutated genes, patients with mutation in *TTN*, *XIRP2*, *PIK3CA*, *TP53*, *MUC16*, *EP300*, *ARID1A*, *ERBB2*, and *KDM6A* had significantly higher TMB (Figure 2A). Previous study has reported that higher TMB indicated a favorable overall survival in bladder cancer patients [17]. Therefore, we performed Kaplan-Meier analysis to identify whether these gene mutations associated with increased TMB were also related to the prognosis of patients with bladder cancer. As shown in Figure 2B, only *EP300* mutation (HR=0.612; 95% CI, 0.399-0.937; P=0.024) was associated with a positive prognosis. However, *EP300* mutation did not remain statistically significance after taking into account age, gender, grade, TNM classification and TMB status in Cox regression model (Table 1), suggesting that *EP300* mutation might not be an independent risk factor of prognosis in bladder cancer patients.

**Table 1:** Univariate and multivariate overall survival analysis of bladder cancer patients by the COX proportional hazards model.

**Factors**	**Univariate**			**Multivariate**	
	**HR (95% CI)**	***P*-value**		**HR (95% CI)**	***P*-value**
Age (year) (<70, ≥70)	1.704 (1.242-2.339)	<0.001		1.765 (1.284-2.425)	<0.001
Gender (male, female)	0.922 (0.650-1.307)	0.648			
Grade (low, high)	21.636 (0.273-1715.747)	0.168			
TNM classification	2.533 (1.672-3.838)	<0.001		2.479 (1.635-3.758)	<0.001
(stage I&II, stage III&IV)					
TMB (low, high)	0.943 (0.915-0.972)	<0.001		0.947 (0.918-0.977)	<0.001
EP300 (wide, mutant)	0.575 (0.338-0.980)	0.042		0.665 (0.385-1.149)	0.144

### Enrichment pathway analysis of *EP300* mutation

**Figure 3:**
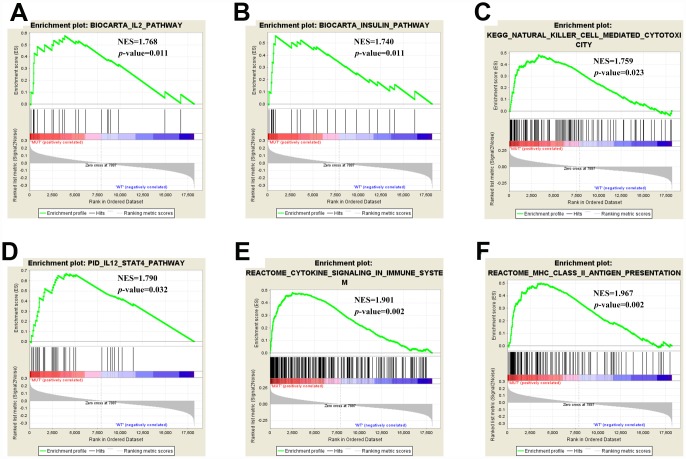
**Significantly enriched pathways associated with *EP300* mutation.** Gene set enrichment analysis has been performed with TCGA. Gene enrichment plots shows that a series of gene sets including (**A**) Biocarta IL2 Pathway, (**B**) Biocarta Insulin Pathway, (**C**) Kegg Natural Killer Cell Mediated Cytotoxicity, (**D**) Pid IL12 Stat4 Pathway, (**E**) Reactome Cytokine Signaling in Immune System, and (**F**) Reactome MHC II Antigen Presentation are enriched in *EP300*-mutant group. NES, normalized enrichment score. The *p-*value is marked in each plot.

As TMB is reported to be a biomarker for immunotherapy and *EP300* mutation was associated with an increased TMB, we further investigated the relation of *EP300* mutation and immune response. GSEA performed with TCGA revealed that Biocarta IL2 Pathway, Biocarta Insulin Pathway, Kegg Natural Killer Cell Mediated Cytotoxicity, Pid IL12 Stat4 Pathway, Reactome Cytokine Signaling in Immune System, and Reactome MHC II Antigen Presentation were significantly enriched in samples with *EP300* mutation (Figure 3A–3F). These findings indicated that samples with *EP300* mutation upregulated signaling pathways involved in immune system.

### Tumor-infiltrating immune cells associated with *EP300* mutation in bladder cancer

**Figure 4:**
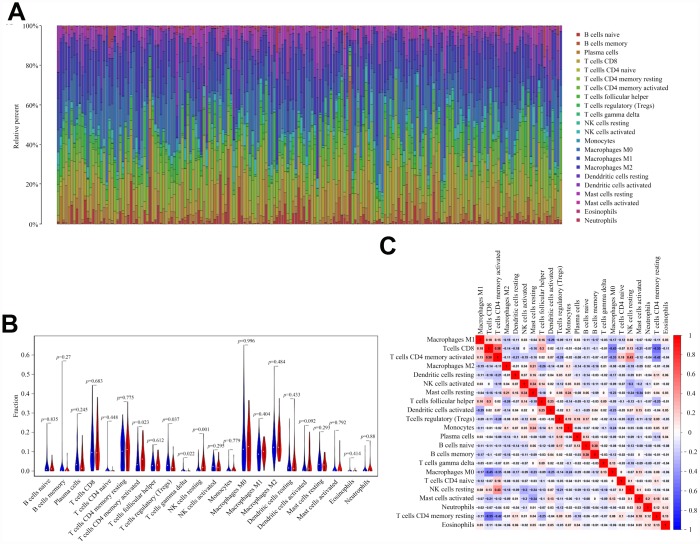
***EP300* mutation is correlated with tumor-infiltrating immune cells.** (**A**) Stacked bar chart shows distribution of 22 immune cells in each sample. (**B**) Violin plot displays the differentially infiltrated immune cells between *EP300*-mutant groups and *EP300*-wild group. Blue color represents *EP300*-wild group, and red color represents *EP300*-mutant group. (**C**) Correlation matrix of immune cell proportions. The red color represents positive correlation and the blue color represents negative correlation.

We next evaluated the association of *EP300* mutation and tumor-infiltrating immune cells in bladder cancer microenvironment using CIBERSORT algorithm. As shown in Figure 4A, the results showed that the composition of 22 immune cell types in each sample varied significantly. Moreover, we observed that activated memory CD4 T cells and resting NK cells were more enriched in *EP300* mutant type group, nevertheless, regulatory T cells (Tregs) and gamma delta T cells were enriched in wild-type group (Figure 4B). Furthermore, the result from the correlation matrix revealed that activated memory CD4 T cells had the strongest positive correlation with CD8 T cells, and also positively correlated with resting NK cells. However, activated CD4 memory T cells had negative correlation with Tregs (Figure 4C).

## DISCUSSION

We respectively characterized the somatic mutation landscape of 412 American bladder cancer samples from TCGA dataset and 101 Chinese bladder cancer samples from ICGC dataset. Then, we found that *EP300* was frequently mutated in both TCGA and ICGC cohorts, and its mutation was associated with higher TMB and favorable clinical prognosis. Meanwhile, samples with *EP300* mutation positive correlated with signaling pathways implicated in immune response. Tumor-infiltrating immune cells results demonstrated that *EP300* mutated samples were more infiltrated in activated memory CD4 T cells and resting NK cells, and less infiltrated in Tregs and gamma delta T cells, which supported the previous findings that such immune cells and pathways play predominant roles in the tumor microenvironment and promote the immune response [18, 19].

*EP300* is known to encode an adenoviral E1A-binding proteins (also known as p300) involved in multiple cellular processes functioning as a transcriptional co-factor and histone acetyltransferase (KAT) as well as its homologous protein partner cAMP response element binding protein (CBP) [20]. *EP300* mutation has been reported in multiple types of human cancer, including breast cancer and squamous cell carcinoma of the head and neck [21, 22]. Specific mutations in *EP300* predict a lower risk for relapses and decrease the mortality of breast cancer [21]. Bladder cancer is a genomically heterogeneous tumor with a high mutation rate, and *EP300* is also a frequently mutated gene in bladder cancer. In our study, we found that mutation in *EP300* indicated a better prognosis and was associated with increased TMB. TMB represents the accumulation of somatic mutations in tumors, and a high TMB contributes to the exposure of more neoantigens, which is likely to elicit a T-cell-dependent immune response [23]. Therefore, we speculated that *EP300* mutation might enhance immune response.

Previous study indicated that as one of the three main KAT families, the p300-CBP family, and p300 in particular, could acetylate and stabilize Forkhead box P3 (Foxp3) protein expression, which was the key transcription factor of Tregs [24]. Tregs are known as adaptive immune cells that maintain self-tolerance and impose a critical check on excessive immune response [25]. Increased Tregs infiltrations are associated with poor outcome in several cancer types, including breast, kidney, and lung cancer [26–28]. Therefore, inhibition of p300 may impair Treg homeostasis and thereby promote antitumor immunity. Recent studies have confirmed that deletion of *EP300* in Tregs suppressed the proliferation and function of Tregs and limited the ability of cancers to avoid immune destruction, thereby decreasing tumor growth [29, 30]. In our study, we also found that bladder cancer samples with *EP300* mutation had lower Tregs loads. Thus, we speculated that *EP300* mutation might impair the function of p300 and affect the generation of Tregs. Moreover, we observed that *EP300* mutation was associated with increased memory CD4 T cells and NK cells infiltrations. The frequency of CD4^+^ lymphocytes with a memory phenotype is reported to significantly increase in patients with tumor free lymph node, and the generation of memory response against tumor antigens and may prevent tumor relapse in patients with different cancers, which predict a longer survival [31, 32]. NK cells as a significant part of innate immune system are characterized by secreting cytokines and cytolytic activity against target cells. Recent studies have shown that NK cells can selectively kill cancer stem cells, which indicate that NK cell-based therapy may become an effective treatment to inhibit tumor relapse and metastasis [33, 34]. Therefore, we found that *EP300* mutation elicited the variation of infiltrated immune cells contributing to antitumor immunity in bladder cancer.

The main limitation in our study is that the ICGC database lacks corresponding clinical data of Chinese bladder cancer, so we cannot verify whether *EP300* mutation is associated with the prognosis of bladder cancer patients in China, and whether it can give rise to the same immune response. Even if *EP300* is also frequently mutated in Chinese bladder cancer sample, the effect may be somewhat heterogeneous between different ethnic groups. As a result, the association of *EP300* mutation and prognosis, including analysis in infiltrated immune cells and signaling pathways need further validation in Chinese bladder samples.

In summary, our study demonstrated that *EP300* was frequently mutated in bladder cancer, and *EP300* mutation was associated with higher TMB and indicated a better prognosis. Furthermore, *EP300* mutation upregulated the signaling pathways of the immune system and evoked an antitumor immune response. These findings reveal a novel gene whose mutation could be served as a biomarker to predict immune response.

## MATERIALS AND METHODS

### Data acquisition

Somatic gene mutations for American bladder cancer samples (n=412) and Chinese bladder cancer samples (n=101) were respectively acquired from TCGA portal (http://portal.gdc.cancer.gov/projects) (up to June 10, 2019) and ICGC portal (http://dcc.icgc.org/ releases/current/Projects) (up to April 3, 2019). Clinical data for 412 bladder cancer samples were downloaded from TCGA. For clinical data, only patients with bladder cancer with complete information (n=402) were included, and those with any missing data concerning survival time, status, age, gender, grade, or TNM classification were excluded.

### Classification of bladder cancer based on TMB

TMB was defined as the number of somatic, coding, indels mutations and base substitution per megabase of genome examined. All base substitutions and idels in the coding region of targeted genes were counted. Silent mutations failing to contribute to an amino acid change were not counted. To calculate the TMB score of each sample, the total number of mutations counted was divided by the exome size (38 megabase (Mb) was used as the estimate of the exome size) [35].

### Bioinformatic analysis

MAF files containing somatic variants for American bladder cancer samples were detected using VarScan and visualized with maftools package [36]. TSV files containing somatic variants for Chinese bladder cancer samples were annotated according to hg19 reference genome and visualized with GenVisR package. Gene set enrichment analysis (GSEA) was performed using Broad Institute GSEA software 3.0 according to previously described [37]. The Gene expression data was downloaded from TCGA portal. Patients were available into two groups according to mutation status of E1A binding protein P300 (*EP300*). The gene set “c2 all.v6.0 symbols.gmt” was downloaded from Molecular Signatures Database (http://software.broadinstitute.org/gsea/msigdb/index.jsp) and was used for the enrichment analysis. Permutations were set to 1000 to obtain a normalized enrichment score (NES). A normal *p*-value <0.05 was considered significantly enriched. CIBERSORT [38], a deconvolution algorithm that evaluates the proportions of 22 tumor-infiltrating lymphocyte subsets in a bulk tumor sample, was used to estimate the relative abundance of immune cell infiltration in patients with different *EP300* status. The number of permutations was set to 1000, and a threshold *p*-value of <0.05 was the criterion for successful computation of a sample.

### Statistical analysis

Statistical analyses were performed with R software (version 3.6.0) and GraphPad Prism 5.0 software (San Diego, CA, USA). Kaplan-Meier survival analysis was used to determine survival curves that reflect the association between gene mutations and prognosis, which were evaluated by the log-rank test. Univariate and multivariate Cox regression analyses were used for survival analysis of clinical characteristics of patients, including age, gender, grade, TNM classification, TMB and *EP300*. Mann-Whitney U test was used to analyze the correlation between mutant genes and TMB. For all comparisons, a two-tailed *p*-value <0.05 was considered statistically significant.
